# More reliable forecasts with less precise computations: a fast-track route to cloud-resolved weather and climate simulators?

**DOI:** 10.1098/rsta.2013.0391

**Published:** 2014-06-28

**Authors:** T. N. Palmer

**Affiliations:** Atmospheric, Oceanic and Planetary Physics, Clarendon Laboratory, Parks Road, Oxford OX1 3PU, UK Oxford Martin Programme on Modelling and Predicting Climate

**Keywords:** weather prediction, climate simulation, stochastic modelling, approximate computing

## Abstract

This paper sets out a new methodological approach to solving the equations for simulating and predicting weather and climate. In this approach, the conventionally hard boundary between the dynamical core and the sub-grid parametrizations is blurred. This approach is motivated by the relatively shallow power-law spectrum for atmospheric energy on scales of hundreds of kilometres and less. It is first argued that, because of this, the closure schemes for weather and climate simulators should be based on stochastic–dynamic systems rather than deterministic formulae. Second, as high-wavenumber elements of the dynamical core will necessarily inherit this stochasticity during time integration, it is argued that the dynamical core will be significantly over-engineered if all computations, regardless of scale, are performed completely deterministically and if all variables are represented with maximum numerical precision (in practice using double-precision floating-point numbers). As the era of exascale computing is approached, an energy- and computationally efficient approach to cloud-resolved weather and climate simulation is described where determinism and numerical precision are focused on the largest scales only.

## 1. Introduction

Today's weather and climate simulators play an increasingly important role in society. Forecasts from weather simulators enable decisions, e.g. on evacuating large numbers of people ahead of the arrival of some extreme tropical cyclone or hurricane. Additionally, climate simulators are now beginning to provide advanced warnings of natural climate variations on seasonal and decadal time scales and are able to provide estimates of how the statistics of weather may be affected by human emission of greenhouse gases into the atmosphere on decadal and longer time scales [1]. However, the truncation scale of weather and climate simulators (*ca* 10 and 100 km, respectively) is still relatively coarse [2], and hence they only provide inaccurate pictures of reality. Current estimates suggest that the ability to bring the resolution of global weather and climate simulators down to convective cloud scales (less than 1 km) will require exascale computing resources [3]. It is unlikely that such computers will be available in operational weather and climate institutes until the 2030s at the earliest (N Wedi 2013, personal communication). Even when such machines are potentially available, it could be that the power requirements of such machines—potentially hundreds of megawatts [4]—could make them unaffordable to weather and climate institutes.

This paper sets out the case for a more energy- and computationally efficient approach to solving the equations for simulating and predicting weather and climate, one in which the conventional hard boundary between the dynamical core and the sets of sub-grid parametrizations is blurred. The reasons why it is both important and urgent to develop such an approach are outlined in §2, where the rationale is discussed for one of the grand challenges in weather and climate prediction science: the development of cloud-resolved simulators. In §3, the traditional approach to solving the partial differential equations (PDEs) of weather and climate, referred to as the ‘canonical numerical ansatz’, is discussed. Because of the observed ‘−5/3’ power-law spectrum, this ansatz cannot be justified rigorously, and alternative approaches are discussed where unresolved processes are represented by simplified stochastic–dynamic systems, rather than deterministic formulae. In §4, it is argued that because high-wavenumber variables in dynamical cores will inherit directly the stochasticity of such closure schemes, we may be significantly over-engineering these dynamical cores by assuming a requirement for determinism and maximum precision, irrespective of scale. An alternative approach to the development of cloud-resolved simulators is outlined, based on variable precision arithmetic and inexact (potentially stochastic) computing hardware.

## 2. Towards cloud-resolved simulators of weather and climate

Before setting out the alternative methodological approach to the canonical numerical ansatz, one might ask: Why bother? The advances made in the last decades have demonstrated the value of existing methodologies in both weather [5] and climate [6,7] simulation. By continuing down the same route, refining numerical methods and parametrizations, and taking advantage of developments in supercomputing technology, surely further advances in skill and realism can be expected in the coming decades?

While advances can indeed be expected, two key questions need to be raised. First, can society afford to wait for these developments to occur? Below it is argued not. Second, the sustained flop rate of current weather and climate simulators on current supercomputers can be around 10% or less of these computers’ peak performance [8]. As we approach the exascale, can we afford to continue to make such relatively poor usage of available computational and (by implication) energy resources?

Consider the first of these questions. Earth's climate is a multi-scale nonlinear dynamical system *par excellence* in which spatially and temporally disparate scales of internal variability of the Earth's climate are strongly coupled. Hence, for example, convectively organized clouds which vary on sub-diurnal time scales are responsible for driving the Madden–Julian oscillation on intraseasonal time scales. In turn, the Madden–Julian oscillation is believed to play an important role in triggering El Niño events on seasonal time scales. Through atmosphere/ocean coupling and atmospheric teleconnections between the tropics and the extratropics, the El Niño event can impact on global temperatures, not only on seasonal to interannual time scales, but also on decadal time scales. For example, the recent ‘hiatus’ in global warming is believed to be linked to deep ocean heat uptake in the tropical Pacific in the aftermath of the unusually strong 1997/98 El Niño event [9,10]. It is possible that the strong warming of the tropical West Pacific associated with this recent hiatus may be responsible for some recent severe weather in the extratropics [11,12].

Numerical models which seek to simulate climate from first principles must attempt to represent as much of this multi-scale dynamics as possible. However, the dynamics of individual convectively organized cloud systems can only begin to be represented in dynamical cores with computational grids of 1 km or less. Given the need to represent (in comprehensive climate simulators) not only the atmosphere, but also the oceans, the cyrosphere and the land surface, an ability to produce ensembles of century-long integrations at 1 km resolution and within reasonable periods of (wall-clock) time (say *ca* one month) will certainly need exaflop computing capability [3]. As the first exaflop machines will not be on the market until the end of the decade, it is unlikely that weather and climate institutes will be able to afford such machines until the 2030s.

Can we afford to wait that long? Let us focus here on the climate issue. Numerical climate simulators are scientific tools which provide key input to climate policy on mitigation and adaptation. While the current generation of climate simulators demonstrates that the threat of dangerous climate change is quite unequivocal, there is considerable uncertainty in the magnitude (and even sign) of some of the feedback processes. Perhaps most important of these are the cloud feedbacks [13]. If cloud feedbacks are strong and positive, humanity is indeed heading for calamitous climate change with business-as-usual emissions. On the other hand, if they are negative, and hence can help offset other feedbacks about whose (positive) sign climate scientists are more confident (water vapour, sea ice, methane release from permafrost, etc.), then human-induced climate change may be something society can largely adapt to. Uncertainty in cloud feedback processes is as large today as it was 30 years ago [14]. Of course, simulating clouds more accurately requires simulating the water cycle more accurately [15], and this in turn requires accurate simulation of the atmosphere and oceanic circulation.

In addition to providing input into climate mitigation policy, global climate simulators have an important role to play in helping society worldwide adapt to climate change. Here the relevant prognostic variables are related to regional changes in precipitation and storminess as much as to temperature. Current generation global climate models provide estimates of changes to such variables; however, our confidence in these estimates is relatively poor, largely because of the relatively poor resolution of global climate models [2].

Finally, reliable regional output from global climate simulators will be of paramount importance if society ever considers seriously the possibility of geoengineering climate, e.g. by spraying aerosols into the stratosphere. A back-of-the-envelope calculation may suggest that such geoengineering will cool the planet and therefore offset human-induced climate change. However, the consequences of such geoengineering on the water cycle are far from clear. These consequences can only be estimated reliably from global climate models which represent the water cycle accurately. As with the issues related to climate adaptation, however, our confidence in being able to estimate regional circulation changes arising from either human-induced climate change or geoengineering is rather poor [15].

## 3. Stochastic parametrization

In Richardson's seminal treatise on weather prediction by numerical process [16], the PDEs governing weather and climate are discretized on a chosen computational grid, with unresolved processes represented by formulae which mimic, for example, molecular viscosity and diffusion. Richardson's use of the concepts of ‘eddy viscosity’ and ‘eddy diffusion’ can be traced back to the work of pioneers in the fluid dynamical theory, such as Boussinesq and Prandtl, and arise from the notion of Reynolds averaging.

Following the development of the electronic digital computer, it was possible to envisage solving these PDEs, using such numerical approximations, faster than the weather itself evolved [17]. The development of comprehensive climate simulators evolved out of such numerical weather prediction simulators [18,19]. Today's weather and climate simulators are unimaginably more complex than those developed by these early pioneers, yet they are still based on the canonical numerical ansatz proposed by Richardson, i.e. dynamical cores, and parametrization formulae which depend on truncation-scale variables from the dynamical core and a number of free parameters [20]. Such a representation is universal, for example, among the simulators contributing to the Coupled Model Intercomparison Project on which the Intergovernmental Panel on Climate Change Working Group 1's Fifth Assessment Report [1] is based.

**Figure 1.**
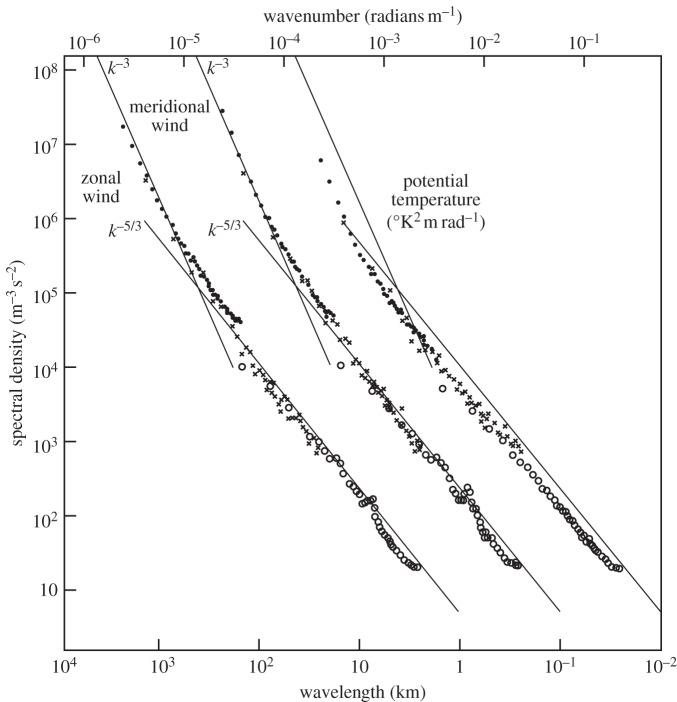
Power spectra of wind and potential temperature based on aircraft observations. The spectra of meridional wind and temperature are shifted by one and two decades to the right, respectively. Lines with slopes −3 and −5/3 are entered at the same relative coordinates for each variable for comparison. (Adapted from [23].)

The PDEs governing weather and climate comprise the Navier–Stokes equations describing conservation of momentum, with corresponding energy and mass conservation equations for evolution of temperature, density and other important variables (such as water in its three phases). As PDEs, these equations couple scales of motion from the planetary scale down to the viscous sub-range. However, it must be recalled that typical truncation scales in weather and climate simulators are many orders of magnitude greater than scales associated with the viscous inertial sub-range. This is an aspect of numerical modelling that has not changed since Richardson's day. Hence, there is no *a priori* reason why, just because the bulk effect of molecules can be parametrized accurately by formulae, the bulk effect of unresolved processes such as convective clouds and sub-grid topography can similarly be parametrized by formulae. Indeed, there are good reasons to suppose that they cannot be so represented [21]. Ultimately, these reasons relate to the approximate self-similar scaling symmetries associated with these PDEs [22]. These symmetries manifest themselves in observational data as power-law structure. For the atmosphere, the most famous of these are the power laws found by Nastrom & Gage [23] (figure 1). On scales less than a few hundred kilometres, the power law shallows from a −3 to a −5/3 slope, consistent with a transition from rotationally dominated to more three-dimensional circulations. The recent book by Lovejoy & Schertzer [24] provides abundant evidence for the pervasive nature of such power-law structure in the atmosphere.

With such power-law structure, sub-grid tendencies will tend to be dominated by individual processes close to the truncation scale. Moreover, as highlighted by [25], in a ‘5/3’ spectrum error doubling times associated with processes at wavenumber *k* will increase with *k*. Hence, tendencies associated with individual processes close to the truncation scale will often be extremely unpredictable and hence only related to the grid scale in some probabilistic sense. Given this, it will be impossible to parametrize sub-grid processes using deterministic formulae based on resolved-scale variables.

In weather and climate institutes, it is commonplace for simulator development to be split between groups that focus on the dynamical core and groups that develop parametrization formulae. The former are concerned with the usual issues in numerical analysis, e.g. reducing discretization and rounding errors, and ensuring good conservation properties. By contrast, the latter are concerned with finding formulae which better describe theoretical understanding of unresolved processes, and trying to fit the values of free parameters arising in such formulae using observations or theory. In the past, improvements [5–7] have arisen by addressing these concerns separately. However, it is less clear that the systematic errors in today's weather and climate simulators (which are typically comparable to the signals such simulators attempt to simulate and predict) can be simply isolated to either the dynamical core or the parametrizations, in the traditional sense. The ‘elephant in the room’ when trying to understand simulator error is the unquantified assumption that we can indeed simulate climate, at levels of accuracy to which we aspire, using the canonical numerical anstatz.

**Figure 2.**
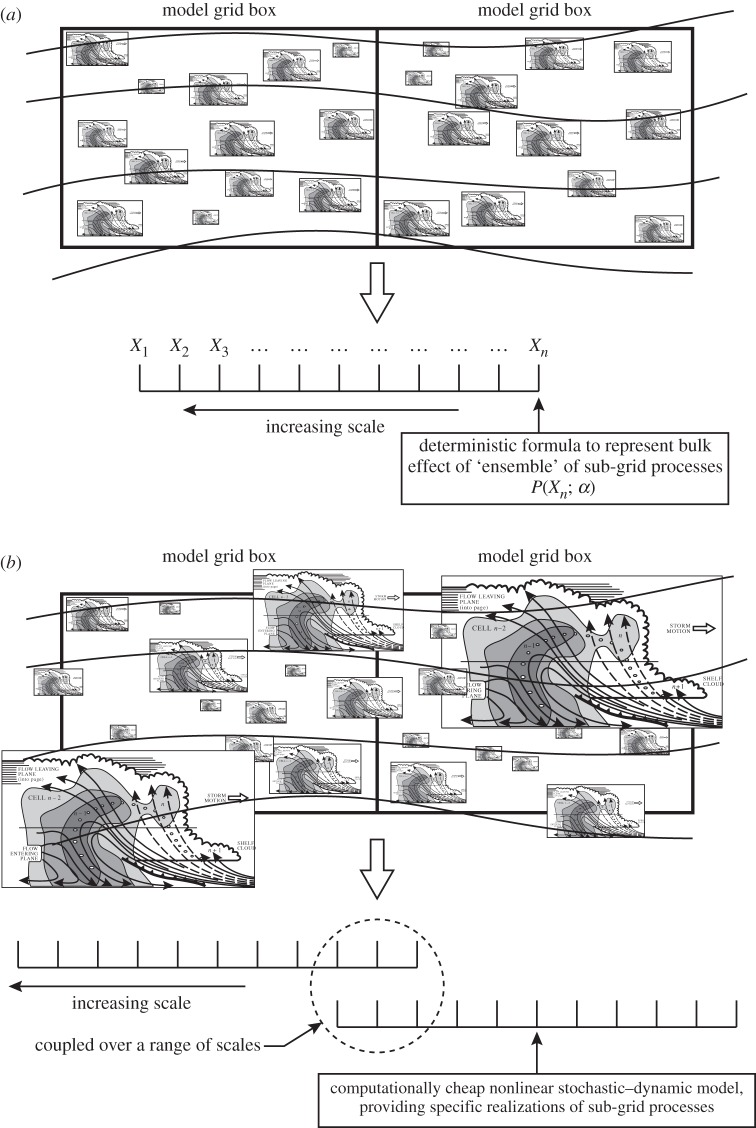
(*a*) Schematic of hypothetical situation where there is some scale separation between resolved and unresolved flow, justifying the notion of deterministic parametrization. (*b*) Schematic of the more realistic situation where there is no scale separation between resolved and unresolved flow, justifying the notion of stochastic parametrization.

Figure 2 shows an alternative approach [21,26,27]. Instead of representing sub-grid-scale processes using formulae, the sub-grid processes are represented by simplified dynamical systems. Such simplified dynamical systems could in principle be represented by purely deterministic dynamics, provided these dynamics were sufficiently chaotic. However, from a computational point of view, it is often much simpler to represent such unpredictability using explicitly stochastic mathematics; not least the statistical behaviour of stochastic systems is much more controllable than the statistical behaviour of deterministic systems. Perhaps the best approach (see examples below) arises from a combination of stochastic and deterministic dynamics. In such a stochastic–dynamic approach, the associated tendencies relate to potential realizations of the sub-grid flow, rather than ensemble-average bulk tendencies. Quantitative justification for such representations can be derived from coarse-grain studies [28,29]. In such studies, one takes output from an integration of a high-resolution (e.g. cloud-resolved limited-area) simulator as ‘truth’. From such an integration, one estimates both exact and parametrized tendencies relative to some coarse-grained grid (e.g. associated with a typical CMIP5 simulator). It is found that sub-samples of exact sub-grid tendencies, conditioned on some small range *Δr* of parametrized tendency, typically show a distribution of values broadly centred on the parametrized range but with standard deviation *σ*≫*Δr*. Moreover, it is found that *σ* increases as the magnitude of the parametrized tendency increases.

Developing this approach, Palmer [21,26] proposed to represent unresolved convective cloud systems using stochastic cellular automata defined on a fine-scale lattice, much smaller than the grid of the dynamical core. Cells are convectively active (‘on’) or inactive (‘off’). The probability of a cell being ‘on’ is determined from grid-scale variables. The (stochastically determined) lifetime of a particular convective cell is increased when nearest neighbours are convectively active; thus, organized convection has a longer lifetime than isolated convection. These organized convective cells can backscatter energy onto the resolved grid [30,31]. Convectively, active cells can be readily advected from one grid box to another by the grid-scale wind using a variant of the ‘Mexican wave’ concept. In this way, a simple representation of sub-grid dynamics can be readily encoded into such a scheme. This concept has been developed into a fully operational stochastic convection scheme by Bengtsson *et al.* [32].

Another lattice-based stochastic approach to the representation of non-interacting convective cloud systems has been developed by Plant & Craig [33]. Here, sub-grid representations of convective systems are represented by draws from Poisson probability distributions—as one would expect from the simple statistical mechanics of non-interacting elements. Khouider *et al.* [34] and Peters *et al.* [35] have generalized these approaches by developing stochastic multi-cloud lattice models, incorporating congestus and stratiform cloud as well as deep convective cloud. The transition between cloud types is determined by Markov chain processes. The development of advective or wave dynamics within such lattice models has yet to be attempted.

Strongly related to this, Shutts & Allen [36] have argued that sub-grid processes could be computed using the correct equations of physics but with reduced complexity and numerical accuracy. These authors draw inspiration from techniques used by computer games animators; these animators are increasingly turning to the proper fluid equations to simulate fluid flow, but with simplified methods (which prevent spurious energy dissipation) for ensuring that the simulations at least look realistic.

Perhaps the sub-grid schemes with the most sophisticated sub-grid dynamics are the so-called superparametrization schemes [37,38]. In such schemes, a full cloud-resolved model (typically projected from three spatial dimensions to two) is incorporated into each grid box. These schemes have shown good performance in simulating key intraseasonal modes of variability [39]. Needless to say, such schemes are more computationally expensive than the more simple cellular automaton or multi-cloud lattice models. Consistent with the remarks above, it seems quite possible that stochastic simplifications can make these superparametrization schemes less computationally demanding [40].

At the other end of the spectrum of stochastic–dynamic sub-grid models is the stochastically perturbed parametrization tendency (SPPT) scheme of the European Centre for Medium-Range Weather Forecasts (ECMWF) [41,42]. In the SPPT scheme, deterministic parametrization tendencies *P* are replaced by a multiplicative noise representation (1+*ϵ*)*P*, where *ϵ* represents a field of Markov chain processes in spherical harmonic space, with prescribed spatio-temporal autocorrelations. This form of multiplicative noise is broadly consistent with results from the coarse-grain budget studies mentioned above. The SPPT scheme has been shown to improve the probabilistic forecast skill of medium-range [42] and seasonal forecasts [43] and to reduce systematic error in the representation of weather regimes [44].

The performance of multiplicative noise parametrizations can be demonstrated using the simple Lorenz 96 model [45],
3.1
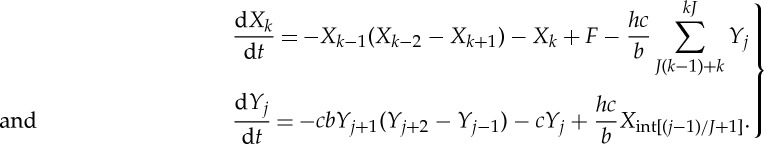


**Figure 3.**
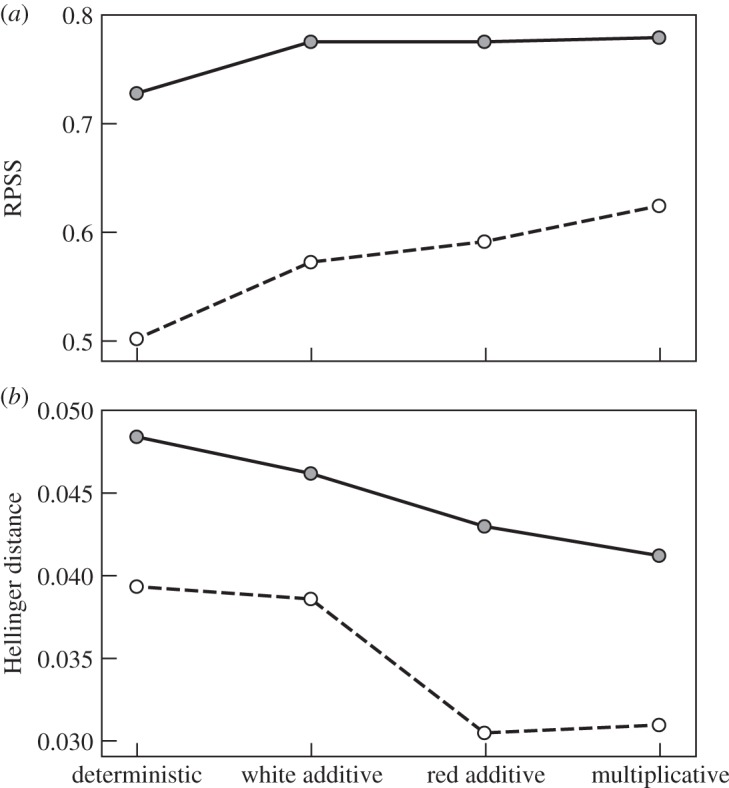
(*a*) Solid and dashed lines show the ranked probability skill scores (RPSS) for 75 initial condition ensemble forecasts at *t*=0.6, based on differences between the Lorenz [47] dynamical system and various parametrized versions of the system, with *c*=10 (solid) and *c*=4 (dashed). (*b*) As (*a*) but for the Hellinger distance between the climatological probability distribution of the Lorenz system and the parametrized versions of the system. (From [45].)

Here, one considers the *X*_*k*_ variables as large scale, and the *Y*_*j*_ variables as small scale. In an environment where the small-scale variables cannot be resolved, we instead solve
3.2
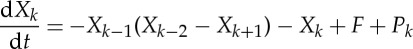
for some parametrized tendencies *P*_*k*_. Wilks [46] has shown that parametrizations with additive noise lead to more skilful probabilistic forecasts than with purely deterministic parametrizations. However, Arnold *et al.* [45] have shown that multiplicative AR1 parametrizations, similar to those of the SPPT scheme, can be even more effective in producing reliable forecasts and accurate climatologies (figure 3).

## 4. Stochastic and imprecise computing

**Figure 4.**
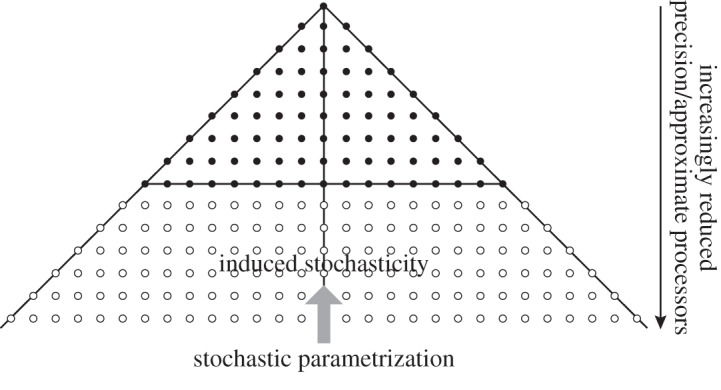
A schematic with each ‘dot’ representing a spherical harmonic coefficient in a triangularly truncated spectral weather or climate simulator. The vertical axis represents total wavenumber, and the horizontal axis zonal wavenumber. The solid dots represent the primary scales of interest, i.e. synoptic and greater scales. If one assumes that the closure problem is inherently stochastic, then the closure process will induce significant stochasticity on the sub-synoptic scales. This implies that the calculations on sub-synoptic scales need not be made with the same level of precision and determinism as those on the synoptic and larger scales.

It has been argued above that the closure problem for weather and climate simulators should be treated as being inherently stochastic, leading to more skilful forecasts and simulations with reduced systematic bias. If one accepts this, then the dynamical cores of climate models will inherit this stochasticity during time integration, especially for variables associated with scales close to the truncation scale. This raises some important questions. What is the real information content in the variables in a dynamical core, as a function of scale? Are we overengineering our dynamical cores (and indeed the parametrizations themselves) by assuming that these variables have more information content than they actually have? If the answer to the latter question is yes, then the following question becomes pertinent. Could we increase the resolution of our dynamical cores by only representing the relevant scale-dependent variables with their appropriate level of information content? Clearly, this approach is going to be most effective in dynamical cores where scale separation is explicit, e.g. in spectral or multi-grid representations (figure 4). (This may provide a rationale for maintaining spectral simulators as the truncation scales reach into the non-hydrostatic domain. It is sometimes said that the Legendre transform for such dynamical cores will be uncompetitive at non-hydrostatic resolutions. However, approximate spherical harmonic transformations have been developed based on exactly the principles discussed in this section: scale-selective precision with the transformations performing precisely on large scales and with degraded performance at small scales [48].)

Two specific issues are explored here. The first can be raised within the framework of the canonical numerical ansatz. For many years now, all dynamical variables in weather and climate prediction models have been represented by double-precision floating-point numbers. Is this necessary for all variables in the dynamical cores and parametrization? For example, representation of variables using single-precision floating-point numbers has some obvious potential advantages: as well as computations potentially taking less time (though this will be a function of chip design) and more numbers being storable in cache, the energy needed to move data from memory to core and from one processor to another can be (approx.) halved if such numbers were represented by single- rather than double-precision floating-point numbers. Both the ECMWF Integrated Forecasting System (P Düben 2013, personal communication) and the Swiss COSMO limited area model (O Führer 2013, personal communication) have been successfully run using single-precision floating-point representations of real number variables. However, as yet there has been no attempt to apply such representations in a scale-selective manner (e.g. as a first step, within the parametrization schemes alone). Again, the relevant issue is real information content. For example, if chip design would allow it, is there merit in representing variables at sufficiently high wavenumbers using half or even quarter precision floating-point numbers? (It can be noted that a cellular automaton, which was advocated above as a way to represent small-scale convective processes in weather and climate models, can be considered a 1/32 precision floating-point number!) If there is a sound rationale for representing variables with varying levels of floating-point precision in comprehensive weather and climate simulators, then this information needs to be communicated to designers of supercomputers.

This raises another important issue concerning the computing hardware on which weather and climate simulators are run. Traditionally, computing has been considered deterministic with computations reproducible to the last bit. However, in [49–52], a new vision for computing has begun to emerge. For a number of reasons, it is becoming increasingly expensive to keep transistors operating deterministically. Not least, the unprecedented increase in the density of chips (the size of a transistor is now getting close to 20 nm) leads to non-deterministic variations in voltage and timing variations in circuits and indeed a chip's reliability can be affected by cosmic ray strikes [53]. The assumption used in hardware design that applications necessarily require correct values for every computation can lead to hardware overdesign whereby so-called guardbands imposed on the hardware result in substantial increased power costs and leave substantial performance potential untapped. In this new vision for energetically super-efficient computing, the software is exposed to such non-determinism. The notion of probabilistic CMOS promoted by Palem [50] attempts to exploit the stochasticity of low-energy circuits as a source of randomness in inherently probabilistic applications.

**Figure 5.**
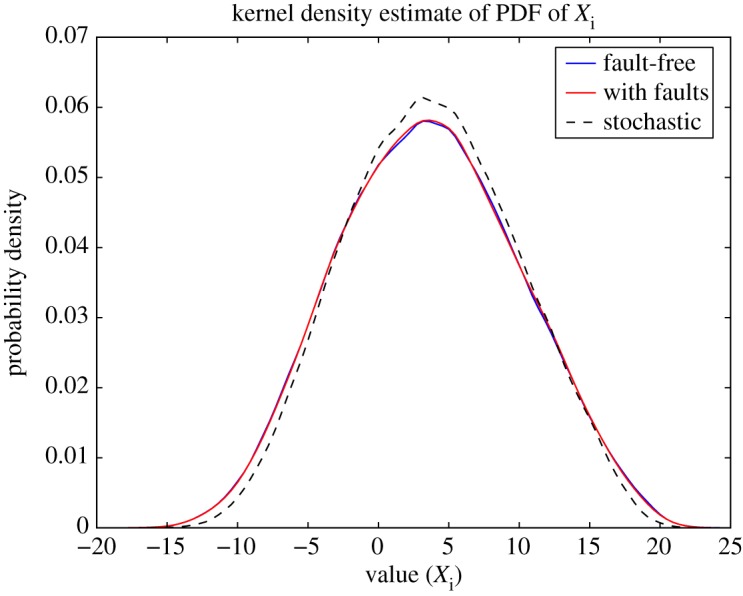
Probability distribution of an *X* variable in the Lorenz 96 model: with a fault-free deterministic computer (blue), with an emulator of an imprecise stochastic computer with 20% error rate in the mantissa of the floating-point real number representation of *Y* variables (red); with multiplicative AR1 stochastic parametrization (dashed). Especially in the print version, the red and blue lines are almost indistinguishable, indicating the very small impact of the use of imprecise processing. (Online version in colour.)

For many applications, it is important to ensure that the software can be made robust to such non-determinism. In the case of weather and climate simulators, such non-determinism can be considered a positive resource. It is interesting to note that the ultimate source of stochasticity in computing hardware is associated with the quantum mechanical noise of electrons moving through the transistors. One could perhaps describe a computer based on such stochastic chip hardware as a quantum computer of the second kind! As with conventional quantum computers, only certain types of computational problem will benefit from this alternative type of technology. It is argued here that weather and climate simulators are representatives of the type of multi-scale computational problem which could benefit from quantum computers of the second kind. In particular, it is possible that both dynamical-core variables *X*(*k*) associated with scales near the truncation scale *k*_*T*_ and all variables in the parametrizations can be represented and manipulated using inexact or stochastic hardware. As an example, we return to the Lorenz 96 model. Figure 5 (from [54]) shows the climatological PDF of one of the large-scale variables *X*_*j*_ for three representations of the Lorenz 96 model. The exact solution is compared with integrations with the best multiplicative noise parametrization (see above) and with an emulation of a stochastic processor, with 10% error on all digits of the mantissa of the floating-point representation of the *Y* variables. (It appears plausible that chip design could focus stochasticity on the mantissa, keeping the exponent error free. According to some estimates allowing 10% error in the mantissa could result in as much as a 90% reduction in power consumption—R Kumar & K Palem 2013, personal communication). The key point is that the climatology of the integration where the *Y* variables are solved on the low-energy chips is almost indistinguishable from the exact solution, and clearly better than the best stochastically parametrized solution. In Düben *et al.* [54], results are shown from a spectral simulator of intermediate complexity, which support the results above.

It may be some years before such stochastic computers have been manufactured and tested. Hence, as an interim measure, the original ideas of inexactness have been adapted to be relevant to existing computing deterministic technologies. In this second approach, errors are induced by eliminating parts of the computing hardware that are not deemed to be especially important to the accuracy of the information being computed. Examples of the application of this ‘probabilistic pruning’ approach to the Lorenz 96 model are described later in this Theme Issue [55].

To conclude this section, some remarks are made on the notion of seamless prediction. The key motivation for seamless prediction studies is that by developing weather and climate simulators that are as similar to one another as possible, insights and constraints from weather time scales (where verification data are relatively plentiful) can be brought to bear on the climate time scale (where verification data are less plentiful). However, in practice, ‘seams’ are inevitably apparent in moving from weather and climate simulators, even though these simulators may share common dynamical cores and parametrizations. Not least is the seam arising from the fact that weather and climate simulators typically have different resolutions. A way to lessen this ‘resolution seam’ may be possible using the ideas presented in this paper. That is to say, instead of changing from high to low resolution as one moves from weather to climate prediction, it may be possible instead to scale-selectively degrade precision with a single fixed resolution. It is planned to test this concept on the two-tier ECMWF ensemble prediction system, where resolution is currently degraded after day 10.

## 5. Conclusion

Since their inception, the PDEs for weather and climate have been solved numerically by projecting the equations onto a computational grid and representing unresolved processes by deterministic formulae. This has been referred to as the canonical numerical ansatz.

**Figure 6.**
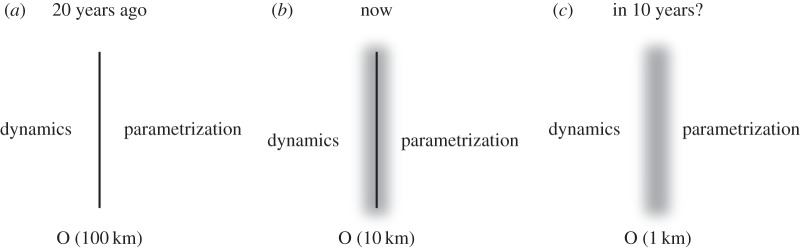
(*a*) For global numerical weather prediction models 20 years ago, there was a clear delineation between the dynamical core and the sub-grid parametrizations with truncation scales around 100 km. (*b*) For global numerical weather prediction models with stochastic parametrizations today, the delineation between dynamical core and sub-grid parametrization is less clear cut. (*c*) It is speculated that in 10 years' time, it may be possible to develop weather and climate simulators where the truncation scale is nominally around 1 km; however, there will be no precise boundary between the dynamical core and the set of stochastic–dynamic parametrizations—instead the relationship between the two will be a blurred concept.

It is suggested here that if we aspire to cloud-resolved weather and climate simulators in the foreseeable future, it may be time to abandon this ansatz as a bedrock of numerical simulation. Instead, a blurring of the boundary between resolved-scale dynamics and sub-grid parametrization is postulated (figure 6). Consistent with this, it is imagined such simulators being integrated on new types of supercomputer which have relatively small numbers of completely bit-reproducible processors, and much larger numbers of energy-efficient approximate or stochastic processors. The metrics of success will necessarily be probabilistic. However, for a chaotic system, which the climate system certainly is, such probabilistic metrics are more appropriate for measuring the quality of forecast skill than more traditional deterministic metrics of forecast skill such as RMS error or anomaly correlation coefficient [27].
